# Clinical features, management and visual outcomes on patients with traumatic hyphema in a reference ophthalmological clinic in Colombia

**Published:** 2020

**Authors:** Virgilio Galvis, Angelica Pedraza-Concha, Alejandro Tello, M. Lina Plata, C. Luis Escaf, D. Ruben Berrospi

**Affiliations:** * Centro Oftalmológico Virgilio Galvis, Floridablanca, Santander, Colombia; ** Fundación Oftalmológica de Santander FOSCAL, Floridablanca, Santander, Colombia; *** Universidad Industrial de Santander, Bucaramanga, Santander, Colombia; **** Universidad Autónoma de Bucaramanga, Floridablanca, Santander, Colombia

**Keywords:** Eye Injuries, hyphema, intraocular pressure, prognosis

## Abstract

**Aim.** To analyze clinical features, treatment, and results of patients with non-penetrating traumatic hyphema in an ophthalmological center in Colombia.

**Methods.** A retrospective cohort study in which medical records of patients with traumatic hyphema were analyzed between 2013 and 2018.

**Results.** 38 eyes of 37 patients (34 men, 3 women) were included. Average age was 30.6 ± 16.6 years. Sports-related (42.1%) and occupational accidents (34.2%) were the main causes. 67.5% of the eyes had grade I hyphema. 95% received topical corticosteroids, 92.1% topical mydriatics and 52.63% ocular hypotensive eyedrops. Two eyes with hyphema grade I did not receive steroids and resolved uneventfully. None of the eyes rebleeded, even without antifibrinolytics. One patient with grade IV hyphema required surgery. Mean hyphema’s clearance time was 8.4 ± 3.2 days. The last mean corrected distance visual acuity was LogMAR 0.25. There were no complications directly related to the hyphema.

**Conclusions.** Working related activities were the second cause of traumatic hyphema in our cohort, which might be attributable to poor awareness of the importance or ocular protection, or limited access to recommended protective devices. Outpatient management enabled adequate outcomes. Corticosteroids and mydriatics were the treatment cornerstone, though seemed not to be imperative when hyphema was grade I. We were not able to support the contributive role from antifibrinolytics, because none of our patients rebleeded in spite of the absence of them.

**Abbreviations:** IOP = intraocular pressure, AC = anterior chamber, CDVA = corrected distance visual acuity

## Introduction

Ocular trauma remains the first cause of hyphema, in which cases, the clinician should look for other associated ocular lesions [**1**-**3**]. 

Although it has a good visual prognosis, uncommonly, traumatic hyphema is directly related to ocular complications such as increased intraocular pressure (IOP) or corneal staining, which may lead to permanent loss of vision. Therefore, the aim of an adequate management (though controversies still exist for traumatic hyphema treatment) is the goal [**1**]. Conservative management options include bed rest, elevation of the head, and eye patching/shielding. In addition, the use of various pharmacological agents has been reported including corticosteroids, antifibrinolytic medication, and cycloplegics. However, apart from the use of antifibrinolytics to prevent rebleeding, there is no clear evidence of the benefit from the use of these conservative or pharmacological measures [**4**]. Surgical interventions are also used to evacuate blood or blood clots from the anterior chamber (AC) in cases of uncontrolled IOP elevation, corneal staining, or persistence of either total or sub-total hyphema [**1**-**4**].

The purpose of the present study was to analyze the clinical features, treatment approaches, and results of a group of patients presenting with traumatic hyphema in a referral ophthalmological center in Colombia. 

## Materials and Methods

The design was a retrospective cohort study including patients who suffered secondary hyphema due to blunt trauma and consulted to a reference ophthalmologic institution in Bucaramanga, northeastern Colombia, between 2013 and 2018. Patients with open globe injuries or spontaneous hyphema were excluded from the study. Data including sex, age, trauma mechanism, timing at first consult, hyphema grade, last corrected distance visual acuity (CDVA), IOP values, timing of the maximum IOP peak, management (including medications or surgery), timing for hyphema reabsorption, rebleeding, and sequelae were collected from medical records and analyzed. Statistical analysis was performed using the Software for Statistics and Data Science STATA. Statistical significance was established as a P value <0.05.

This study followed the tenets of the Declaration of Helsinki and the Institutional Ethical Committee granted approval for its development.

## Results

38 eyes (23 right eyes and 15 left eyes) from 37 patients (34 men/3 women), were included in the analysis of the following variables at the moment of the initial examination: age, sex, trauma mechanism, IOP, CDVA. Only 25 patients (26 eyes, due to one case of bilateral compromise after blunt trauma with a car’s airbag) continued attending follow-up visits for at least one week. The mean follow-up period of these 26 eyes was 1.4 months +/- 1.6 (0.27–6.7). The average age of presentation was 30.6 ± 16.6 years (2.2-67.3 years). The patients consulted between 36.6 ± 47.2 hours after the trauma (range 1 to 192 hours). Grade of hyphema at the initial consultation were: microhyphema (Grade 0): 4 eyes (10.8%), Grade I: 25 eyes (67.5%), Grade II: 2 eyes (5.4%), Grade III: 0 eyes (0%) and Grade IV: 5 eyes (13.51%). In two cases (5.1%), the grade of hyphema was not described.

None of the eyes rebleeded and only one patient, with grade IV hyphema, required surgery (AC washout) during the follow-up period due to persistent hyphema and very high IOP (42 mmHg). The mean time of hyphema’s clearance in the group of 22 eyes that initially had macroscopically visible hyphema (i.e. grade I to IV) who did not undergo AC washout, and had a follow-up time for at least one week, was approximately 8.4 ± 3.2 days (considering the moment of resolution as the follow-up visit when the examiner recorded the total reabsorption of the hyphema).

No eye patches were used. From the whole group of 38 eyes, 36 eyes (94.7%) received topical corticosteroids, 35 eyes (92.1%) topical mydriatics and 20 eyes (52.63%) ocular hypotensive eyedrops. Those before mentioned two eyes that did not receive steroids had a grade I hyphema, which reabsorbed on the 4th day. From the patients who consulted our institution, none received antifibrinolytic agents (systemic or topic).

We could not identify clinically significant complications possibly related to the hyphema itself, and not directly related to the trauma per se. In one case, originally presenting grade I hyphema in both eyes, a mild deposition of hematic cells and pigment were seen at the last visit (six weeks after the trauma) in both eyes, though without affecting the CDVA (this case is discussed in more detail below). Other observed complications among the total group of 38 eyes, were considered to be related with the blunt trauma rather than with the hyphema: corneal opacity/edema in 4 eyes (10.5%), angular recession in 5 eyes (13.2%), glaucoma in 1 eye (2.6%), crystalline lens subluxation in 1 eye (2.6%), posterior capsule opacification in 4 eyes (10.5%), post-traumatic uveitis in 3 eyes (7.9%), vitreous hemorrhage in 4 eyes (10.5%) and retinal contusion in 6 eyes (15.8%). 

The mechanisms of the trauma that caused the hyphema are shown in **Table 1**. Sports-related (42.1%) and occupational accidents (34.2%) were the main causes. There were no significant differences in frequency between these two groups (p=0.4725). On the other hand, violence (5.3%) and road accidents (5.3%) showed a significantly lower rate as cause of these traumas related to hyphema, than sports (p=0.0002) and work (p= 0.0016) accidents.

**Table 1:** Mechanism of the trauma in cases of traumatic hyphema

	Number of eyes	(% of eyes)
Sports/ games related	16	42,1%
Soccer ball	11	
Sticks	2	
Paintball	1	
Rubber band	1	
Direct hit	1	
Work related	13	34,2%
Brushcutter	6	
Bottle cap	2	
Steam burst	1	
Stick	1	
Chopping stone	1	
Elastic rope	2	
Violence	2	5,3%
Rocks	2	
Road traffic issue	2	5,3%
Airbag*	2	
Other** (fireworks, motorbike handlebar, soda cap)	5	13,2%
Total eyes	38	
*One patient with bilateral blunt globe trauma due to airbag trauma		
**Non labor related activities		

Regarding IOP (**Table 2**), 21 out of 26 eyes with follow-up time longer than one week, had the information at the first examination with a mean of 18.1 ± 11.9 mmHg. 3 eyes (14.3%) had an IOP higher than 30 mmHg initially, all of them with hyphema grade I. In addition to those 3 eyes, other 2 eyes that firstly showed hyphema grade IV, had an IOP peak higher than 30 mmHg during the follow-up period (2 to 6 days after the trauma). At the last follow-up visit, only one eye, different from those 5 eyes already mentioned, had an IOP higher than 20 mmHg (23 mmHg in spite of ocular hypotensive eye drops).

Excluding one eye with previous visual acuity of No Light Perception (related to an old retinal detachment), the last CDVA was LogMAR 0.25 (Snellen 20/35.6), with a range from LogMAR 0.0 (Snellen 20/20) to LogMAR 1.52 (Snellen 20/662). The four eyes with CDVA lower than 20/50 at the last follow up visit (mean 1.1 ± 1.3 months) had other comorbidities related to the trauma, which explained the diminished vision (corneal edema, retinal edema, vitreous hemorrhage, inflammatory pupillary membrane). 

**Table 2:** IOP in eyes with at least 1 week of follow-up

	Initial (n=21 eyes)		Maximum peak (n=22)		Final (n=25)	
IOP (mmHg)	Eyes	%	Eyes	%	Eyes	%
0-10	5	23,8%	1	4,5%	3	12,0%
11-20	12	57,1%	11	50,0%	21	84,0%
21-30	1	4,8%	5	22,7%	1	4,0%
31-40	1	4,8%	2	9,1%	0	0,0%
≥41	2	9,5%	3	13,6%	0	0,0%

## Discussion

Trauma is a frequent cause of ophthalmological emergency consults [**5**,**6**]. Around two thirds of traumatic hyphema cases are attributable to blunt trauma, between 30 and 60% of them in different series, occurring during sports practicing [**3**,**7**]. The aforementioned data are congruent with our findings because sports related blunt trauma represented the first cause, though it is relevant to mention that trauma related to work activities represented the second most frequent cause in our cohort. Unfortunately, it was not established if these patients were wearing appropriate eye protection. This is undoubtedly a critical point for future studies. On the other hand, in children, as expected, most of the causes have been found to be related to sports or games related accidents and to domestic accidents [**8**,**9**]. In the present study, seven patients younger than 18 years old were included: 4 cases had sports related trauma, 1 case was related to fireworks but two were related to violence (hit by stones thrown by other people).

Hyphema grading system was done according to the level of the hemorrhage in the AC. Micro-hyphema corresponds to hardly seen floating red blood cells in the AC; grade I to an occupied volume by visible blood of the AC of less than one third; grade II when more than a third but lesser than half of the volume of the AC is full with blood; grade III when more than half, but not the total volume of the AC is occupied, and grade IV or “Eight-ball” (or as suggested by Bansal et al., “red-ball”) hyphema when the hemorrhage reaches the total filling of the AC [**1**]. Also, atypical hyphemas have been reported as linear trans-pupillary radial hyphemas [**10**].

Visual prognosis in traumatic hyphema depends on several factors, including trauma mechanism, grade of hyphema, additional globe or ocular annexes injuries and visual acuity right after blunt trauma [**1**-**3**]. In the absence of other concomitant injuries in the globe, it is rare for traumatic hyphema to cause permanent visual loss [**1**-**4**]. Rocha et al. mentioned that a poor visual prognosis was owed to posterior segment lesions [**8**]. Simanjunktak et al. also related coexisting intravitreal hemorrhage, cataract, iridodialysis, choroidal rupture, higher grade of hyphema, poorer initial visual acuity, and late consultation after the onset of injury with a poorer visual prognosis [**2**].

Reported incidence of rebleeding varies in a wide range from 0 to 38% [**4**,**8**,**9**,**11**,**12**]. There were no cases of rebleeding in our cohort.

Historical studies have shown that the risk of an increase in IOP is directly related to the degree of the hyphema being approximately 10% in the cases of grade I and II, approximately 25% in grade III and between 50% and 100% in hyphema grade IV. On the other hand, incidence rates of secondary glaucoma vary from 45 to 67% after a rebleeding episode [**1**,**13**,**14**]. In the present study, three out of four eyes with hyphema grade IV showed an IOP peak higher that 21 mmHg (two had 34 mmHg and one had 42 mmHg). In addition, other two eyes with hyphema grade I had IOP peaks higher than 30 mmHg (32 and 42 mmHg), possibly related to concomitant injuries to the AC angle structures directly related to the trauma. 

Other potential complication of hyphema is permanent corneal hematic staining. Corneal bloodstaining might be accompanied by endothelial dysfunction and corneal decompensation [**1**,**15**]. No case in the series showed corneal bloodstaining, but one patient with bilateral trauma and hyphema grade I had mild blood and pigment deposits on the corneal endothelium in both eyes. Possibly, the particular mechanism of trauma (car’s airbag) caused this adherence of the red blood cells to the endothelium.

Regarding therapeutic options, there is a discussion on the need of using topical corticosteroids in traumatic hyphema cases [**1**,**4**,**9**,**12**]. In a retrospective study including 206 patients, Türkoğlu et al. found that patients who were treated with topical corticosteroids, were not less likely to experience a rebleeding or a poor visual outcome than those treated with supportive therapy alone [**11**]. 95% of our cohort received steroids; meanwhile two cases did not receive and still reabsorbed the totality of hyphema in 4 days with no complications. However, these two eyes had only grade I hyphema. 

Hospitalization of the patients for several days have been frequently reported [**2**,**4**,**11**,**12**,**16**-**18**]. In contrast, we managed them as outpatients with regular controls, similarly as some other studies [**4**,**8**,**9**,**12**,**19**,**20**]. As mentioned, in our outpatient managed cohort, none of the patients rebleeded. Therefore, we agreed with the conclusions of Gharaibeh et al. and several other authors, that hospitalization and strict bed rest should only be advocated for patients considered to be at high risk of rebleeding, noncooperation, with sickle cell trait/disease or with other systemic comorbidities justifying the admission to the hospital [**4**,**8**,**9**,**12**,**19**-**21**].

The time of the primary hemorrhage clearance has been reported between 2.7 and 4.5 days [**4**,**8**,**9**,**12**,**18**]. In the present study, it was considered to be approximately 8.4 ± 3.2 days, which seemed to be longer than the previously reported studies. However, since the patients were not seen on a daily basis and the study design was retrospective, it was not possible to determine the exact time of resolution, so the best approximation was done according to the description of hyphema resolution in the medical record. However, at that moment, the hyphema could have disappeared one or some days before. This is a weakness of this study.

Furthermore, we did not find any significant complication considered as directly caused by the presence of hyphema. One patient who had bilateral ocular trauma related to an airbag in a traffic accident showed mild hematic and pigment deposits on the endothelium in both eyes and on the anterior capsule of the lens in the right eye. Initially, he had bilateral hyphema grade I, which resolved in less than 1 week, though he also had corneal abrasions and edema in both eyes. His final DCVA was 20/20 in both eyes. It is possible that the specific mechanism of the trauma, with a forceful compression of the cornea against the iris, made the pigment and red blood cells adhere more easily to the endothelium.

Gharaibeh et al. recently performed a meta-analysis including 20 randomized and 7 quasi-randomized studies (2643 patients in total) and found no evidence of a positive effect on final visual acuity by any of the medical interventions that they evaluated, which practically included all the non-pharmacological and pharmacological approaches published. In addition, they concluded that the use of aminocaproic acid compared with no use, seemed to increase the duration of hyphema, but, on the other hand, reduced the rate of recurrent hemorrhage [**4**]. In our cohort, none of the patients received antifibrinolytic agents (similarly to several other published studies) [**9**,**10**,**13**] and no case of rebleeding was reported in the medical records, therefore we were not able to compare results regarding rebleeding rate and reabsorption timing related with antifibrinolytic usage.

A weakness of the present study was that the race of the patients was not registered. However, in our region, the vast majority of the population is mestizo (Hispanic) and the proportion of African Americans is minimal. It is important however, to emphasize that it is necessary to rule out sickle cell disease or trait in any patient with African ancestry presenting with traumatic hyphema [**1**,**4**,**21**].

Sports related activities in the present study were in accordance with other reports, the most frequent cause for traumatic hyphema. However, our second most reported cause were the work-related activities. This could be due to poor awareness of prevention measures while performing such tasks or limited access to recommended protective devices. Therefore, we consider imperative to develop educational campaigns in order to highlight the need of using appropriate protective spectacles or face shields when the practice of sports or the performance of work activities may expose the eyes to some risk of trauma.

In the present study, without neither in-hospital treatment nor eye patching, only one patient out of 25 (with at least one week of follow-up) required a surgical procedure, and we did not identify any complication directly related to the presence of hyphema. Concerning pharmacological approach, corticosteroids and mydriatics were used in the majority of the patients, though they seemed not imperative in hyphema grade I, as demonstrated in two cases in this study. We were not able to support the mentioned contributive role from antifibrinolytics on preventing rebleeding, because none of our patients presented rebleeding even though none received any type of topic or systemic antifibrinolytic.

**Sources of funding**

None.

**Disclosure**

The authors declare no conflicts of interest.
